# Preoperative prediction of HER2 expression and sentinel lymph node status in breast cancer using a mammography radiomics model

**DOI:** 10.3389/fonc.2025.1578458

**Published:** 2025-06-04

**Authors:** Ziqian Zhao, Hongyi Yuan, Xinyu Song, Wen Liu, Yanyan Chen, Xiaoli Wang, Chao Dong, Binlin Ma

**Affiliations:** ^1^ The Clinical Medical Research Center of Breast and Thyroid Tumor in Xinjiang, Tumor Hospital Affiliated to Xinjiang Medical University, Urumqi, China; ^2^ Department of Breast Surgery, First People’s Affiliated Hospital of Xinxiang Medical University, Xinxiang, China; ^3^ Department of Artificial Intelligence and Smart Mining Engineering Technology Center, Xinjiang Institute of Engineering, Urumqi, China

**Keywords:** breast cancer, HER2 expression, sentinel lymph node, mammography, radiomics

## Abstract

**Background:**

This study aimed to develop and validate radiomic features derived from mammography (MG) to differentiate between various HER2 expression types (HER2-positive, HER2-low, and HER2-zero) and to preoperatively assess sentinel lymph node (SLN) status in breast cancer.

**Methods:**

A retrospective analysis was conducted using clinicopathological and imaging data from 838 female breast cancer patients diagnosed at the Affiliated Tumor Hospital of Xinjiang Medical University between January 2016 and September 2024. The patients were randomly divided into a training set (n=586) and a test set (n=252) in a 7:3 ratio. Multivariate logistic regression analysis identified independent clinical predictors. Tumor segmentation and radiomic feature extraction were performed on mammography images. The least absolute shrinkage and selection operator (LASSO) method was applied for feature selection, and the radiomics model was developed. Model performance was assessed using the area under the receiver operating characteristic curve (AUC), calibration curve, and decision curve analysis.

**Results:**

There were no significant differences in clinicopathological factors and mammographic features between the training and test sets (P>0.05). Multivariate analysis identified ethnicity, lesion size, vascular tumor thrombus, clinical stage, tumor margin, and HER2 expression as independent predictors for SLN metastasis. Lesion size, PR expression, menopausal status, SLN metastasis, Ki67, CK5/6 expression, and calcification were independent predictors for HER2 expression. The SLN metastasis prediction model achieved AUCs of 0.84 in the training set and 0.83 in the test set. The HER2 expression model showed AUCs of 0.87 (positive), 0.82 (low), and 0.85 (zero) in the training set, and 0.84 (positive), 0.78 (low), and 0.84 (zero) in the test set.

**Conclusion:**

Radiomic features based on mammography can effectively preoperatively predict SLN status and HER2 expression types in breast cancer, offering valuable insights for individualized treatment strategies.

## 1 Introduction

According to the 2024 Global Cancer Statistics, breast cancer remains the most commonly diagnosed malignancy among women, with an estimated 2.41 million new cases and 670,000 deaths worldwide. In China, approximately 420,000 new cases were reported, accounting for 18.2% of all female cancers, and the 5-year survival rate varies from 82% in early-stage to 28% in metastatic disease (1). Modern oncology integrates surgery, radiotherapy, and systemic therapies (e.g., targeted drugs and immunotherapies), yet challenges persist in balancing efficacy with invasiveness (2). Future directions emphasize minimally invasive diagnostics and precision medicine, as outlined in recent studies (3). Axillary lymph node metastasis is one of the important features of breast cancer and has a key impact on the staging, diagnosis, treatment and prognosis of breast cancer. Sentinel lymph node biopsy (SLNB) has gradually replaced Axillary lymph node dissection (ALND) and has become the preferred method for clinical evaluation of patients with axillary lymph node negative early breast cancer (4). However, SLNB has limitations, including a high false-negative rate, procedural invasiveness, excessive lymph node resection, and risks of complications, prolonged operative time, and elevated costs. It is also limited by medical conditions and doctor’s operating level. Some hospitals are not yet equipped to carry out SLNB (5). Consequently, there is pressing demand for the development of effective, non-invasive methods capable of predicting sentinel lymph node metastasis (SLNM) in breast cancer, which would significantly contribute to reducing surgical trauma and improving diagnostic accuracy.

With the continuous deepening of the concept of precision medicine, breast cancer treatment is increasingly developing in the direction of individualized and multidisciplinary comprehensive intervention. Studies have shown that about 20% to 30% of breast cancer patients have positive of the human epidermal growth factor receptor 2 (HER2) gene (6, 7). Past targeted drugs have only targeted HER2-positive breast cancer patients and have limited efficacy in HER2-negative patients. However, in the HER2-negative population, approximately 45% to 55% of patients have HER2-Low expression (8, 9). Recent research (10, 11) shows the advent of a new antibody-drug conjugate (ADC) has given HER2-Low breast cancer patients new treatment opportunities in preoperative neoadjuvant treatment.

The early and accurate identification of HER2 gene status plays a pivotal role in implementing personalized treatment strategies and optimizing prognosis (12). Currently, HER2 status is assessed through surgery, biopsy, or genetic analysis, but these methods are limited by tissue sample size and tumor heterogeneity, leading to high false-negative rates and inconsistent results. Thus, establishing a simple, non-invasive strategy to assess both sentinel lymph node and HER2 status in breast cancer is essential for improving diagnostic accuracy and reducing surgical risks.

Breast imaging examination methods include ultrasound, mammography, MRI, CT, etc., each of which has its own advantages and disadvantages. Ultrasound assessment of axillary lymph nodes is more convenient and cost-effective, but image quality depends largely on the experience level of the operator, which may lead to fluctuations in accuracy and reliability. In contrast, mammography has achieved a high degree of standardization and is relatively less affected by operations, so the image quality is more stable (13). With the rapid development of artificial intelligence technology, in recent years, many studies have used radiomic models to detect lesions, distinguish benign and malignant tumors, predict molecular typing of breast cancer, assess axillary lymph node metastasis risk and predict prognosis, and have achieved high diagnostic efficiency (14, 15). At present, most studies based on radiomic methods to predict HER2 status of breast cancer use MRI images, mainly targeting HER2 positive and negative. Only a few studies involve HER2-Low status, and there is still a lack of molybdenum target radiomic studies that can accurately distinguish different HER2 states (16–18).

This study aims to combine preoperative mammography images with clinical and pathological data to create a radiomics model that predicts sentinel lymph node metastasis (SLNM) and HER2 status. The goal is to provide reliable, non-invasive diagnostic evidence for preoperative breast cancer evaluation, axillary lymph node metastasis risk assessment, and personalized treatment planning.

## 2 Materials and methods

### 2.1 Study subjects

This retrospective study included 838 female breast cancer patients who received treatment at Xinjiang Medical University Cancer Hospital between January 2016 and September 2024. The patients met the following inclusion criteria: (1) aged 24 to 88 years; (2) pathologically confirmed breast cancer; (3) sentinel lymph node biopsy (SLNB) or axillary lymph node dissection (ALND), with complete ALND if SLN was positive; (4) complete immunohistochemistry (IHC) data (ER, PR, HER2); (5) preoperative mammography; (6) unilateral breast cancer diagnosis in women aged 20–88 years; (7) complete clinical, pathological, and mammographic data; (8) no prior endocrine therapy, radiotherapy, or chemotherapy; (9) signed informed consent. Exclusion criteria included: (1) incomplete clinical data or poor-quality mammography; (2) no postoperative HER2 IHC or fluorescence *in situ* hybridization (FISH) testing, or an IHC score of 2+ without FISH; (3) distant metastasis; (4) prior breast cancer or other malignancies; (5) male breast cancer.

### 2.2 Clinical data collection

The 838 patients were randomly divided into a training set (586 cases) and a validation set (252 cases) in a 7:3 ratio. Data collected included age, ethnicity, menopausal status, lesion size, histological grade, TNM stage, vascular tumor thrombus, ER, PR, HER2, Ki-67, CK5/6, nerve invasion, SLN metastasis, and mammographic features (e.g., breast density, mass shape, margin, density, architectural distortion, calcification, skin changes). Ethnicity was classified into four categories: Han Chinese, Uyghur, Kazakh, and Others (including Hui and Mongolian). This classification reflects the predominant ethnic groups in Xinjiang and aligns with prior epidemiological studies in this region. HER2 status was classified according to the 2018 ASCO/CAP guidelines as HER2-zero (IHC score of 0), HER2-low (IHC score of 1+ or 2+ with negative FISH), or HER2-positive (IHC score of 3+ or 2+ with positive FISH) (19).

### 2.3 Instruments and methods

#### 2.3.1 Mammography image acquisition

IHC or FISH serves as the gold standard for HER2 assessment in breast cancer. Experienced breast X-ray specialists analyzed craniocaudal (CC) and mediolateral oblique (MLO) images using standard imaging techniques, ensuring maximum compression and automatic exposure control. Particular attention was given to lesions, axillary lymph nodes, and skin conditions during image acquisition. Image omics feature extraction and analysis.

All image data were processed using Unet software for segmentation. The region of interest (ROI) was manually outlined by a radiologist with over five years of experience, unaware of the pathological results. Using the Python-based pyradiomics toolkit, 1,409 features were initially extracted. After stability screening with an intra-class correlation coefficient (ICC > 0.75), 1,302 highly stable features were retained for subsequent analysis. Features included first-order statistics, 2D shape descriptors, texture features, and high-order features (e.g., GLCM, GLRLM, GLSZM, GLDM, NGTDM). Synthetic Minority Over-sampling Technique (SMOTE) was used to balance data. Z-score normalization was applied to standardize feature values across the training cohort, using the mean and standard deviation of each feature derived from the training set, which were then applied to both training and test sets to avoid data leakage. Feature selection was performed using interclass correlation coefficient (ICC), independent sample t-test, and LASSO, with features having ICC > 0.75 retained. The data were split into training and test cohorts (7:3), with t-tests used to identify statistically significant features, followed by LASSO screening.

#### 2.3.2 Model construction

A support vector machine (SVM) algorithm was employed to model the features through LASSO. For the SVM implementation, we employed a radial basis function(RBF)kernel with y parameter scaling, while reserving 99% of the dataset for training through a test_size parameter of 0.01.For LASSO regression, we implemented 10-fold cross-validation with a values spanning 4 logarithmic scales(1074 to 101),maximum iterations of 100,000,and random state stabilization(seed=15). Class balancing was achieved through weighted samples, with LASSO regularization strength optimized across three orders of magnitude using 10-fold cross-validation and a convergence tolerance of 1e-4. The performance of models was evaluated using receiver operating characteristic (ROC) curves, calibration curves, and clinical value assessed using decision curve analysis (DCA).

### 2.4 Statistical analysis

Statistical analysis was performed using SPSS 21, R (version 3.4.1), and Python (version 3.1). All tests were two-sided, with P < 0.05 considered significant. Quantitative data are presented as mean ± standard deviation (X ± s); for normally distributed data, independent t-tests were used, while non-parametric Mann-Whitney U tests were applied to ranked data. Chi-square tests were used for categorical data comparisons. Ethnicity was treated as a categorical variable. Chi-square tests compared distributions between groups, and multivariate logistic regression included ethnicity as dummy variables (Set the Han Chinese as the reference group). Univariate and multivariate logistic regression analysis identified clinical and imaging features associated with HER2 expression and SLN metastasis. ROC and DCA curves were generated using Python, with 95% confidence intervals (CI). The model’s performance was evaluated by area under the curve (AUC), sensitivity, and specificity.

## 3 Results

### 3.1 Analysis of clinical characteristics of sentinel lymph node metastasis

This study included 838 patients (mean age 52.4 ± 10.6 years), 413 of whom had sentinel lymph node metastasis and 425 did not, all with complete clinical and pathological data. No significant differences were found between the training and test groups for any clinical or pathological factors (P > 0.05) (**Table 1**). In the training cohort, univariate analysis identified 12 clinical and pathological factors associated with sentinel lymph node status, including ethnicity, lesion size, histological grade, vascular tumor thrombus, nerve invasion, menopausal status, Ki67, clinical stage, mass margin, mass density, skin changes, and HER2 expression. These 12 factors were analyzed using multivariate logistic regression, which identified six independent risk factors for sentinel lymph node metastasis: ethnicity, lesion size, vascular tumor thrombus, clinical stage, tumor margin, and HER2 expression (**Table 2**).

**Table 1:** Baseline characteristics of the study sample.

Baseline parameters	Total (n = 838)	Training (n=588)	Testing (n = 250)	*P*
HER2 expression				0.360
HER2 zero expression	246 (29.356)	164 (27.891)	82 (32.800)	
HER2 low expression	297 (35.442)	213 (36.224)	84 (33.600)	
HER2 positive expression	295 (35.203)	211 (35.884)	84 (33.600)	
Sentinel lymph node metastasis				0.975
No sentinel lymph node metastasis	425 (50.716)	298 (50.680)	127 (50.800)	
Sentinel lymph node metastasis	413 (49.284)	290 (49.320)	123 (49.200)	
Age (years)				0.201
>40	739 (88.186)	524 (89.116)	215 (86.000)	
≤40	99 (11.814)	64 (10.884)	35 (14.000)	
nationality				0.491
Han	511 (60.979)	363 (61.735)	148 (59.200)	
minority	327 (39.021)	225 (38.265)	102 (40.800)	
Lesion size (cm)				0.997
>2	523 (62.411)	367 (62.415)	156 (62.400)	
≤2	315 (37.589)	221 (37.585)	94 (37.600)	
Histological grading				0.197
I	36 (4.296)	30 (5.102)	6 (2.400)	
II	399 (47.613)	280 (47.619)	119 (47.600)	
III	403 (48.091)	278 (47.279)	125 (50.000)	
Vascular aneurysm thrombus				0.955
none	515 (61.456)	361 (61.395)	154 (61.600)	
have	323 (38.544)	227 (38.605)	96 (38.400)	
ER expression (%)				0.975
>10%	482 (57.518)	338 (57.483)	144 (57.600)	
≤10%	356 (42.482)	250 (42.517)	106 (42.400)	
PR expression (%)				0.739
>20%	366 (43.675)	259 (44.048)	107 (42.800)	
≤20%	472 (56.325)	329 (55.952)	143 (57.200)	
Nerve invasion				0.443
no	732 (87.351)	517 (87.925)	215 (86.000)	
yes	106 (12.649)	71 (12.075)	35 (14.000)	
Whether menopause				0.862
no	453 (54.057)	319 (54.252)	134 (53.600)	
yes	385 (45.943)	269 (45.748)	116 (46.400)	
Ki67(%)				0.501
>20%	617 (73.628)	429 (72.959)	188 (75.200)	
≤20%	221 (26.372)	159 (27.041)	62 (24.800)	
CK5/6				0.658
Negative	622 (74.224)	439 (74.660)	183 (73.200)	
Positive	216 (25.776)	149 (25.340)	67 (26.800)	
Clinical staging				0.818
I	224 (26.730)	162 (27.551)	62 (24.800)	
II	439 (52.387)	307 (52.211)	132 (52.800)	
III	152 (18.138)	103 (17.517)	49 (19.600)	
IV	23 (2.745)	16 (2.721)	7 (2.800)	
Breast density				0.877
Density	59 (7.041)	40 (6.803)	19 (7.600)	
Heterogeneous density	649 (77.446)	458 (77.891)	191 (76.400)	
Fatty	130 (15.513)	90 (15.306)	40 (16.000)	
Lump shape				0.929
Round/spherical	85 (10.143)	60 (10.204)	25 (10.000)	
Lobular/Irregular	753 (89.857)	528 (89.796)	225 (90.000)	
Edge of the tumor				0.965
smooth	118 (14.081)	83 (14.116)	35 (14.000)	
glitch	720 (85.919)	505 (85.884)	215 (86.000)	
Mass density				0.780
Isodensity	247 (29.475)	175 (29.762)	72 (28.800)	
High Density	591 (70.525)	413 (70.238)	178 (71.200)	
Structural distortion				0.565
none	368 (43.914)	262 (44.558)	106 (42.400)	
have	470 (56.086)	326 (55.442)	144 (57.600)	
Calcification				0.455
none	164 (19.570)	119 (20.238)	45 (18.000)	
have	674 (80.430)	469 (79.762)	205 (82.000)	
Skin changes				0.605
none	754 (89.976)	527 (89.626)	227 (90.800)	
have	84 (10.024)	61 (10.374)	23 (9.200)	

**Table 2:** Univariate and multivariate logistic regression analysis for predicting SLNM.

Subgroups	Univariable Analysis					Multivariable Analysis				
	β	SE	Z	P	OR (95%CI)	β	SE	Z	P	OR (95%CI)
age	0.01	0.21	0.04	0.964	1.01 (0.66 ~ 1.54)					
nationality	0.56	0.14	3.93	<.001	1.75 (1.33 ~ 2.32)	0.39	0.19	2.02	0.044	1.48 (1.01 ~ 2.17)
Lesion size	0.88	0.15	5.97	<.001	2.40 (1.80 ~ 3.21)	-1.05	0.26	-3.98	<.001	0.35 (0.21 ~ 0.59)
Histological grading	1.41	0.41	3.41	<.001	4.09 (1.82 ~ 9.19)	0.66	0.56	1.18	0.238	1.93 (0.65 ~ 5.74)
Vascular aneurysm thrombus	1.90	0.16	11.78	<.001	6.66 (4.86 ~ 9.13)	1.45	0.20	7.13	<.001	4.27 (2.86 ~ 6.36)
ER expression	0.24	0.14	1.74	0.082	1.28 (0.97 ~ 1.68)					
PR expression	0.19	0.14	1.34	0.180	1.21 (0.92 ~ 1.58)					
Nerve invasion	1.09	0.23	4.77	<.001	2.97 (1.90 ~ 4.64)	0.37	0.30	1.23	0.218	1.45 (0.80 ~ 2.63)
Menopausal status	-0.30	0.14	-2.18	0.029	0.74 (0.56 ~ 0.97)	0.21	0.19	1.12	0.262	1.24 (0.85 ~ 1.80)
Ki67 expression	0.32	0.16	2.02	0.043	1.38 (1.01 ~ 1.87)	-0.15	0.24	-0.61	0.540	0.86 (0.54 ~ 1.38)
CK56 expression	-0.24	0.16	-1.49	0.136	0.79 (0.58 ~ 1.08)					
Clinical staging	3.21	0.52	6.13	<.001	24.76 (8.88 ~ 69.07)	3.22	0.61	5.25	<.001	25.00 (7.51 ~ 83.23)
Breast density	-0.13	0.27	-0.48	0.634	0.88 (0.52 ~ 1.50)					
Lump shape	0.36	0.23	1.57	0.116	1.44 (0.91 ~ 2.27)					
Edge of the tumor	1.31	0.23	5.71	<.001	3.69 (2.36 ~ 5.79)	1.05	0.29	3.55	<.001	2.85 (1.60 ~ 5.07)
Mass density	0.32	0.15	2.08	0.038	1.37 (1.02 ~ 1.85)	0.22	0.21	1.02	0.310	1.24 (0.82 ~ 1.89)
Structural distortion	0.22	0.14	1.58	0.114	1.25 (0.95 ~ 1.64)					
Calcification	0.30	0.18	1.71	0.088	1.35 (0.96 ~ 1.90)					
Skin changes	1.24	0.26	4.73	<.001	3.46 (2.07 ~ 5.79)	0.58	0.35	1.69	0.091	1.79 (0.91 ~ 3.54)
HER2 expression	0.58	0.18	3.30	<.001	1.79 (1.27 ~ 2.53)	0.61	0.24	2.58	0.010	1.84 (1.16 ~ 2.92)

OR, Odds Ratio, CI, Confidence Interval.

### 3.2 Analysis of clinical characteristics of HER2 expression

Of the 838 patients, 246 (29.4%) had HER2-zero (0), 297 (35.4%) had HER2 1+ or 2+ (FISH-), and 295 (35.2%) had HER2 2+ (FISH+) or HER2 3+. No significant differences were found between the training and test groups (P > 0.05). In the training cohort, after incorporating 20 variables into univariate and multivariate logistic regression, seven factors were identified as independent risk factors for HER2 expression: lesion size, PR expression, menopausal status, SLN metastasis, Ki67, CK5/6 expression, and calcification (**Table 3**).

**Table 3:** Univariate and multivariate logistic regression analysis for predicting HER2 positive, low, and zero expression.

Subgroups	Univariable Analysis					Multivariable Analysis				
	β	SE	t	P	OR (95%CI)	β	SE	t	P	OR (95%CI)
age	-0.18	0.20	-0.90	0.370	0.84 (0.57 ~ 1.24)					
nationality	0.07	0.13	0.57	0.569	1.08 (0.83 ~ 1.39)					
Lesion size	0.40	0.13	3.08	0.002	1.50 (1.16 ~ 1.94)	0.40	0.18	2.26	0.024	1.48 (1.05 ~ 2.09)
Histological grading	0.65	0.32	2.06	0.039	1.92 (1.03 ~ 3.55)	0.05	0.35	0.16	0.876	1.06 (0.53 ~ 2.10)
Vascular aneurysm thrombus	0.20	0.13	1.54	0.123	1.22 (0.95 ~ 1.58)					
ER expression	0.25	0.13	1.92	0.055	1.29 (0.99 ~ 1.66)					
PR expression	-0.28	0.13	-2.23	0.026	0.75 (0.59 ~ 0.97)	-0.46	0.15	-3.03	0.002	0.63 (0.47 ~ 0.85)
Nerve invasion	0.20	0.19	1.04	0.299	1.22 (0.84 ~ 1.79)					
Menopausal status	-0.29	0.13	-2.26	0.024	0.75 (0.58 ~ 0.96)	-0.32	0.13	-2.42	0.015	0.72 (0.56 ~ 0.94)
Sentinel lymph node status	0.75	0.13	5.82	<.001	2.13 (1.65 ~ 2.74)	0.78	0.16	4.80	<.001	2.19 (1.59 ~ 3.01)
Ki67 expression	0.65	0.14	4.66	<.001	1.92 (1.46 ~ 2.54)	0.55	0.16	3.41	<.001	1.74 (1.26 ~ 2.39)
CK56 expression	-0.67	0.15	-4.50	<.001	0.51 (0.38 ~ 0.68)	-0.94	0.17	-5.60	<.001	0.39 (0.28 ~ 0.54)
Clinical staging	0.75	0.20	3.80	<.001	2.11 (1.44 ~ 3.11)	-0.39	0.28	-1.38	0.166	0.68 (0.39 ~ 1.18)
Breast density	-0.23	0.26	- 0.88	0.381	0.80 (0.48 ~ 1.33)					
Lump shape	-0.10	0.20	-0.51	0.610	0.90 (0.60 ~ 1.35)					
Edge of the tumor	0.02	0.18	0.13	0.895	1.02 (0.72 ~ 1.46)					
Mass density	-0.23	0.14	-1.66	0.097	0.79 (0.60 ~ 1.04)					
Structural distortion	0.13	0.13	1.00	0.315	1.14 (0.88 ~ 1.46)					
Calcification	0.59	0.16	3.73	<.001	1.81 (1.32 ~ 2.47)	0.41	0.16	2.51	0.012	1.51 (1.10 ~ 2.09)
Skin changes	0.05	0.21	0.25	0.804	1.05 (0.70 ~ 1.58)					

OR, Odds Ratio, CI, Confidence Interval.

### 3.3 Construction of radiomics prediction model for sentinel lymph node metastasis

**Figure 1:**
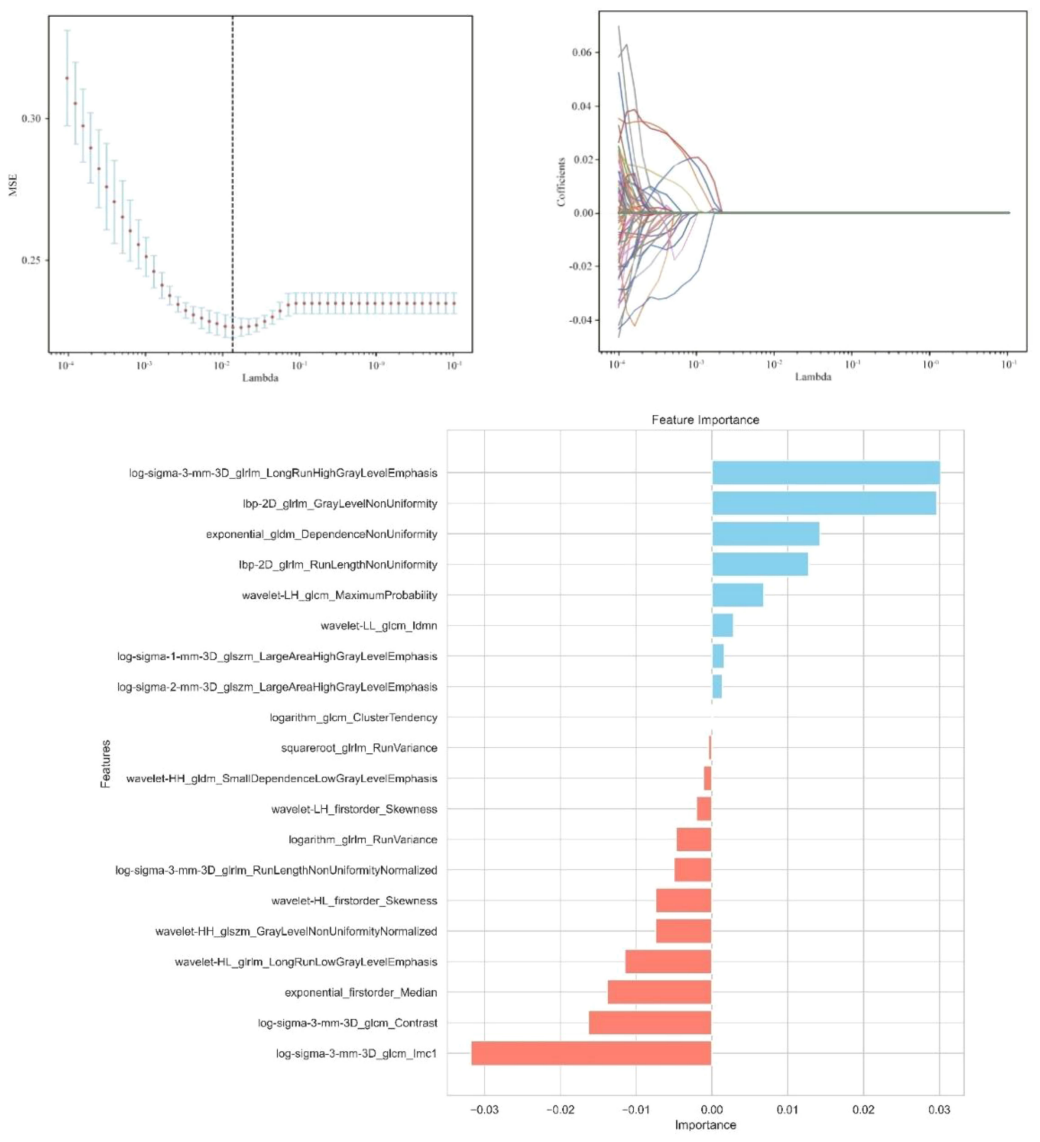
Independent sample t-test and LASSO regression analysis were used to screen the significant features for predicting sentinel lymph nodes.

**Figure 2:**
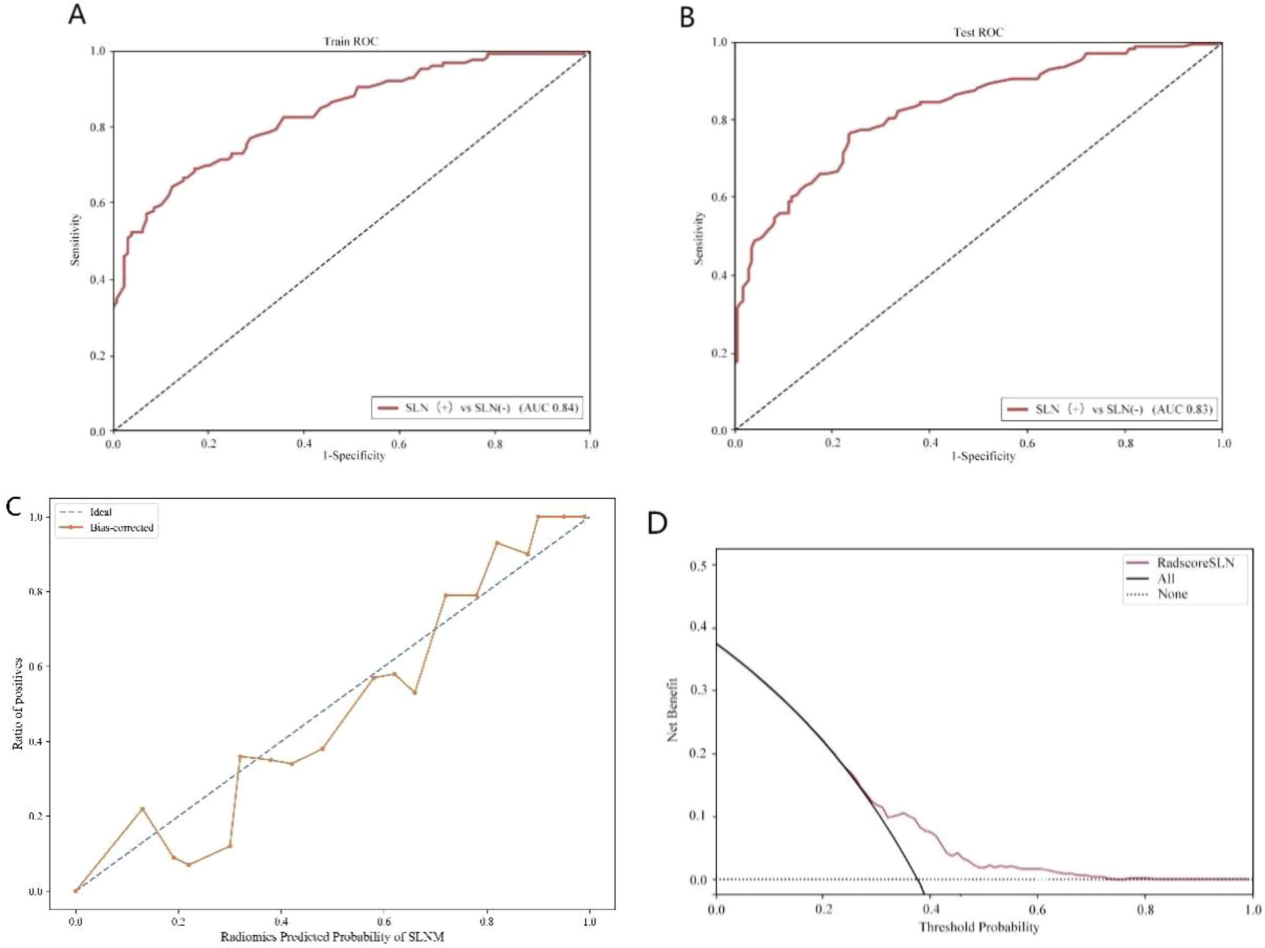
Sentinel lymph node status prediction model. **(A)**: Training set ROC curve **(B)**: Test set ROC curve **(C)**: Calibration curve analysis of prediction model **(D)**: Decision curve analysis of prediction model. Training Queue AUC (95% CI): 0.84 (0.79-0.87); Testing Queue AUC (95% CI): 0.83 (0.71-0.84).

A total of 1,302 stable radiomic features (ICC ≥ 0.75) were analyzed for each patient, with 1,060 showing high stability (ICC ≥ 0.75). After preliminary screening with an independent sample t-test, the LASSO algorithm selected the 20 best features (**Figure 1**)(**Table 4**). These features include: First Order Statistics (3 features), GLCM features (4 features), GLSZM features (3 features), GLRLM features (5 features), GLDM features (2 features), Wavelet Transform Features (4 features), Multi-scale Filtering Features (5 features), LBP Features (2 features). Based on these features and their weighting coefficients, a radiomics score (radscore) was calculated for each patient. The support vector machine (SVM) algorithm was used to construct a prediction model. The model’s performance was evaluated using the receiver operating characteristic (ROC) curve, achieving an AUC of 0.84 in the training set and 0.83 in the validation set (**Figure 2**) (**Table 5**).

**Table 4:** Optimal characteristics for predicting SLNM.

Optimal Characteristics	P value
original_firstorder_Kurtosis	<0.01
original_firstorder_Skewness	0.03
original_glszm_HighGrayLevelZoneEmphasis	0.04
original_glszm_LargeAreaHighGrayLevelEmphasis	<0.01
original_ngtdm_Busyness	<0.01
wavelet-LH_firstorder_Maximum	0.02
wavelet-LH_firstorder_Median	<0.01
wavelet-LH_glszm_HighGrayLevelZoneEmphasis	0.02
wavelet-LH_glrlm_RunEntropy	<0.01
wavelet-LH_glrlm_RunLengthNonUniformityNormalized	0.01
wavelet-LH_ngtdm_Contrast	0.01
wavelet-HL_glcm_Imc1	0.02
wavelet-HL_glszm_GrayLevelNonUniformity	0.04
wavelet-HL_glrlm_ShortRunLowGrayLevelEmphasis	0.04
wavelet-HL_gldm_DependenceVariance	0.01
wavelet-HH_glszm_HighGrayLevelZoneEmphasis	0.02
wavelet-HH_glszm_LargeAreaLowGrayLevelEmphasis	0.03
wavelet-HH_gldm_DependenceEntropy	0.04
wavelet-LL_glszm_SizeZoneNonUniformity	0.03
log-sigma-1-mm-3D_glszm_SizeZoneNonUniformity	0.04
log-sigma-2-mm-3D_firstorder_Mean	0.04
log-sigma-2-mm-3D_glcm_MCC	<0.01
log-sigma-2-mm-3D_glcm_MaximumProbability	0.01
log-sigma-2-mm-3D_glszm_GrayLevelNonUniformityNormalized	0.04
log-sigma-2-mm-3D_glszm_SizeZoneNonUniformity	<0.01
log-sigma-2-mm-3D_glszm_SmallAreaLowGrayLevelEmphasis	0.02
log-sigma-2-mm-3D_ngtdm_Strength	0.01
log-sigma-3-mm-3D_glszm_GrayLevelNonUniformityNormalized	0.04
log-sigma-3-mm-3D_glszm_SmallAreaLowGrayLevelEmphasis	0.01
log-sigma-3-mm-3D_glrlm_LongRunLowGrayLevelEmphasis	<0.01
log-sigma-3-mm-3D_ngtdm_Contrast	<0.01
square_gldm_DependenceNonUniformity	0.03
squareroot_ngtdm_Contrast	0.04
logarithm_firstorder_InterquartileRange	0.02
logarithm_firstorder_Maximum	0.01
logarithm_firstorder_Median	<0.01
logarithm_glcm_MaximumProbability	0.02
logarithm_glcm_SumSquares	<0.01
logarithm_glszm_GrayLevelNonUniformityNormalized	<0.01
logarithm_glrlm_ShortRunLowGrayLevelEmphasis	<0.01
exponential_firstorder_Maximum	0.04
exponential_gldm_DependenceNonUniformity	0.04
exponential_gldm_GrayLevelNonUniformity	0.02
exponential_gldm_SmallDependenceLowGrayLevelEmphasis	0.04
gradient_glszm_GrayLevelVariance	0.02
gradient_glrlm_ShortRunLowGrayLevelEmphasis	<0.01
gradient_ngtdm_Contrast	0.04
gradient_ngtdm_Strength	<0.01
lbp-2D_firstorder_Median	<0.01
lbp-2D_glrlm_GrayLevelNonUniformity	0.04
lbp-2D_glrlm_RunLengthNonUniformity	0.03
lbp-2D_glrlm_RunLengthNonUniformityNormalized	0.04
lbp-2D_glrlm_RunVariance	0.04
lbp-2D_gldm_DependenceEntropy	0.04

**Table 5:** Performance evaluation of sentinel lymph node status model.

Model Evaluation	Training Queue				Testing Queue			
	AUC (95% CI)	Sensitivity	Specificity	Accuracy	AUC (95% CI)	Sensitivity	Specificity	Accuracy
Performance evaluation of sentinel lymph node status models	0.84 (0.79-0.87)	0.73	0.76	0.75	0.83 (0.71-0.84)	0.73	0.76	0.79

### 3.4 Construction of radiomics prediction model for HER2 expression

**Figure 3:**
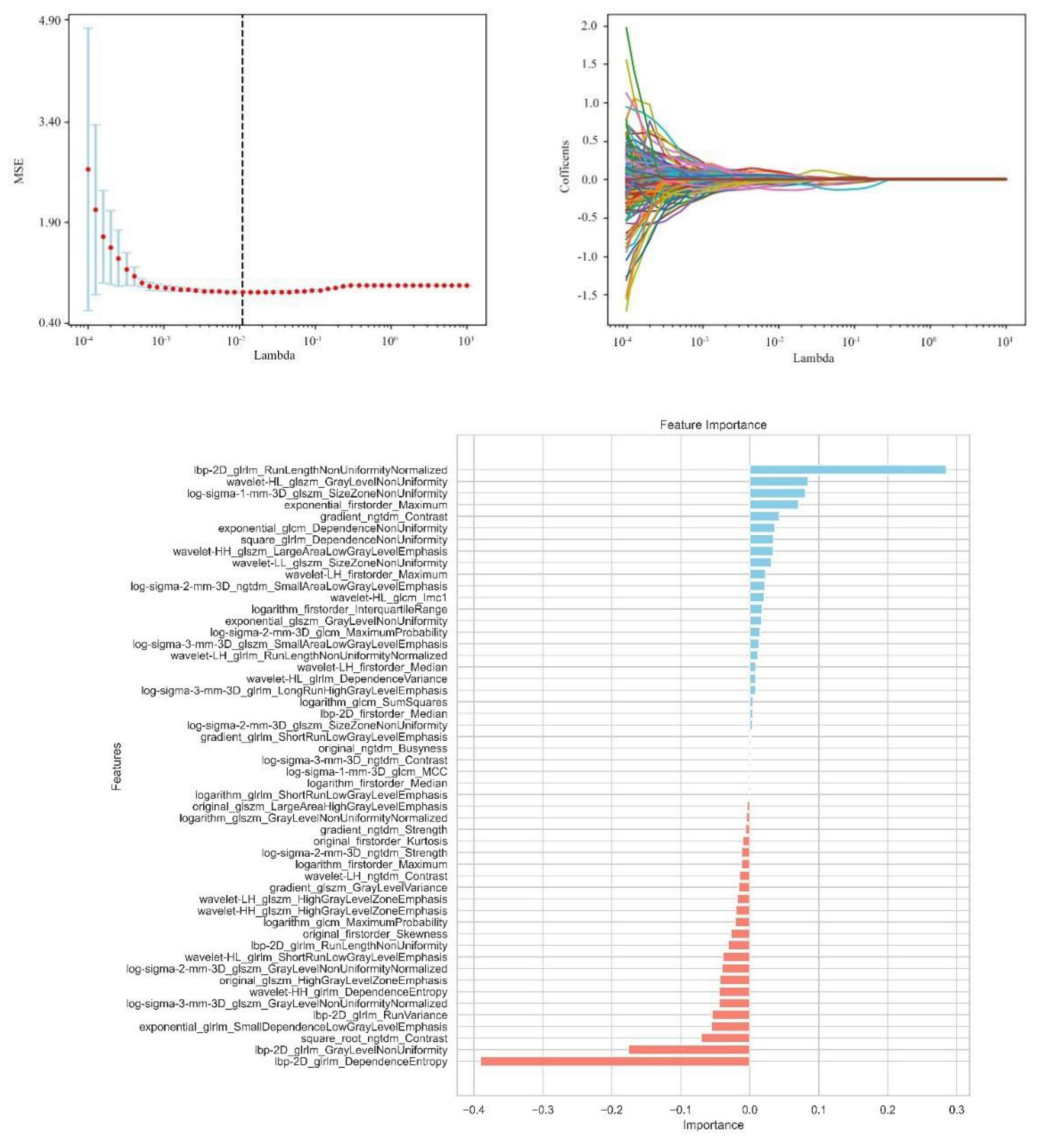
Independent sample t-test and LASSO regression analysis were used to screen significant features for predicting HER2.

**Figure 4:**
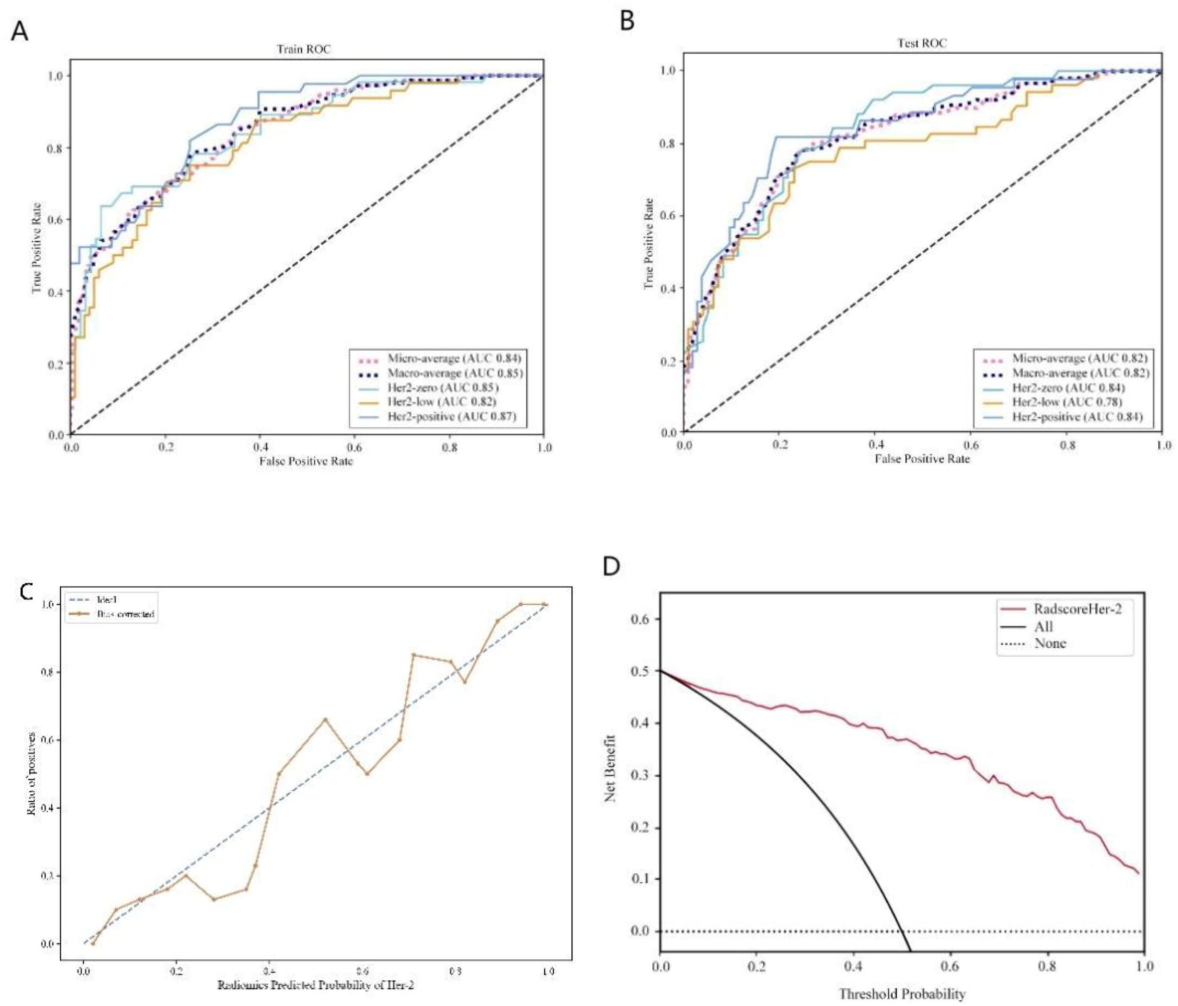
HER2 expression prediction model. **(A)**: Training set ROC curve **(B)**: Test set ROC curve **(C)**: Calibration curve analysis of prediction model **(D)**: Decision curve analysis of prediction model. Training Queue AUC (95% CI): Her2-zero 0.85 (0.74~ 0.87), Her2-low 0.82 (0.72-0.86), Her2-positive 0.87 (0.73-0.93); Testing Queue AUC (95% CI): Her2-zero 0.84 (0.75 ~ 0.89), Her2-low 0.78 (0.61 ~ 0.86), Her2-positive 0.84 (0.76–0.89).

From the craniocaudal (CC) and mediolateral oblique (MLO) mammographic images, 1,302 radiomic features were extracted. After dimensionality reduction using ICC and t-tests, 54 optimal features were selected by LASSO regression (**Figure 3**) (**Table 6**). These features include: First Order Statistics (9 features), GLCM features (5 features), GLSZM features (10 features), GLRLM features (8 features), GLDM features (6 features), Wavelet Transform Features (12 features), Multi-scale Filtering Features (10 features), LBP Features (4 features). A radiomics model based on these features showed strong prediction performance for HER2 status. ROC analysis revealed AUCs of 0.85 (training) and 0.84 (validation) for HER2-zero (0); 0.82 (training) and 0.78 (validation) for HER2 1+ or 2+ (FISH-); and 0.87 (training) and 0.84 (validation) for HER2 2+ (FISH+) or HER2 3+ (**Figure 4**) (**Table 7**).

**Table 6:** Optimal characteristics for predicting HER2 positive, low, and zero expression.

Optimal Characteristics	P value
original_firstorder_Kurtosis	<0.01
original_firstorder_Skewness	0.03
original_glszm_HighGrayLevelZoneEmphasis	0.04
original_glszm_LargeAreaHighGrayLevelEmphasis	<0.01
original_ngtdm_Busyness	<0.01
wavelet-LH_firstorder_Maximum	0.02
wavelet-LH_firstorder_Median	<0.01
wavelet-LH_glszm_HighGrayLevelZoneEmphasis	0.02
wavelet-LH_glrlm_RunEntropy	<0.01
wavelet-LH_glrlm_RunLengthNonUniformityNormalized	0.01
wavelet-LH_ngtdm_Contrast	0.01
wavelet-HL_glcm_Imc1	0.02
wavelet-HL_glszm_GrayLevelNonUniformity	0.04
wavelet-HL_glrlm_ShortRunLowGrayLevelEmphasis	0.04
wavelet-HL_gldm_DependenceVariance	0.01
wavelet-HH_glszm_HighGrayLevelZoneEmphasis	0.02
wavelet-HH_glszm_LargeAreaLowGrayLevelEmphasis	0.03
wavelet-HH_gldm_DependenceEntropy	0.04
wavelet-LL_glszm_SizeZoneNonUniformity	0.03
log-sigma-1-mm-3D_glszm_SizeZoneNonUniformity	0.04
log-sigma-2-mm-3D_firstorder_Mean	0.04
log-sigma-2-mm-3D_glcm_MCC	<0.01
log-sigma-2-mm-3D_glcm_MaximumProbability	0.01
log-sigma-2-mm-3D_glszm_GrayLevelNonUniformityNormalized	0.04
log-sigma-2-mm-3D_glszm_SizeZoneNonUniformity	<0.01
log-sigma-2-mm-3D_glszm_SmallAreaLowGrayLevelEmphasis	0.02
log-sigma-2-mm-3D_ngtdm_Strength	0.01
log-sigma-3-mm-3D_glszm_GrayLevelNonUniformityNormalized	0.04
log-sigma-3-mm-3D_glszm_SmallAreaLowGrayLevelEmphasis	0.01
log-sigma-3-mm-3D_glrlm_LongRunLowGrayLevelEmphasis	<0.01
log-sigma-3-mm-3D_ngtdm_Contrast	<0.01
square_gldm_DependenceNonUniformity	0.03
squareroot_ngtdm_Contrast	0.04
logarithm_firstorder_InterquartileRange	0.02
logarithm_firstorder_Maximum	0.01
logarithm_firstorder_Median	<0.01
logarithm_glcm_MaximumProbability	0.02
logarithm_glcm_SumSquares	<0.01
logarithm_glszm_GrayLevelNonUniformityNormalized	<0.01
logarithm_glrlm_ShortRunLowGrayLevelEmphasis	<0.01
exponential_firstorder_Maximum	0.04
exponential_gldm_DependenceNonUniformity	0.04
exponential_gldm_GrayLevelNonUniformity	0.02
exponential_gldm_SmallDependenceLowGrayLevelEmphasis	0.04
gradient_glszm_GrayLevelVariance	0.02
gradient_glrlm_ShortRunLowGrayLevelEmphasis	<0.01
gradient_ngtdm_Contrast	0.04
gradient_ngtdm_Strength	<0.01
lbp-2D_firstorder_Median	<0.01
lbp-2D_glrlm_GrayLevelNonUniformity	0.04
lbp-2D_glrlm_RunLengthNonUniformity	0.03
lbp-2D_glrlm_RunLengthNonUniformityNormalized	0.04
lbp-2D_glrlm_RunVariance	0.04
lbp-2D_gldm_DependenceEntropy	0.04

**Table 7:** Performance evaluation of HER2 expression model.

Model Evaluation	Training Queue				Testing Queue			
	AUC (95% CI)	Sensitivity	Specificity	Accuracy	AUC (95% CI)	Sensitivity	Specificity	Accuracy
HER2 zero expression	0.85 (0.74~ 0.87)	0.84	0.73	0.82	0.84 (0.75 ~ 0.89)	0.86	0.77	0.75
HER2 low expression	0.82 (0.72-0.86)	0.78	0.73	0.78	0.78 (0.61 ~ 0.86)	0.75	0.71	0.76
HER2 positive expression	0.87 (0.73-0.93)	0.87	0.84	0.81	0.84 (0.76–0.89)	0.84	0.78	0.81

## 4 Discussion

The evolving approach to breast cancer surgery emphasizes minimizing trauma and enhancing patient quality of life. Accurate preoperative assessment of HER2 status and sentinel lymph node metastasis is crucial for tailoring treatment plans, evaluating prognosis, and predicting recurrence risk (20, 21). This study demonstrates that radiomic features derived from mammography effectively predict HER2 expression subtypes and sentinel lymph node (SLN) metastasis. Our model achieved AUCs of 0.82–0.87 in the training set, outperforming traditional clinical-pathological assessments. These findings provide a non-invasive tool to guide personalized treatment strategies and reduce unnecessary surgical interventions. The decision curve analysis (DCA) further confirmed the clinical utility of the model, showing a net benefit across a wide range of threshold probabilities (10–60%). For example: In high-risk patients (predicted SLN metastasis probability >60%), clinicians may prioritize SLNB to confirm metastasis and plan axillary dissection, aligning with current guidelines. Conversely, in low-risk patients (predicted probability <20%), the model supports avoiding unnecessary SLNB procedures, opting instead for watchful waiting or non-invasive monitoring. This stratification could reduce surgical complications by 30–40% in low-risk cohorts, as observed in breast cancer risk management studies (21).This dual-threshold approach highlights the utility of DCA in translating model outputs into actionable decisions, as similarly demonstrated in glioma biomarker research (22). Unlike ROC analysis, which evaluates diagnostic accuracy (AUC), DCA quantifies clinical net benefit by balancing true-positive gains against false-positive harms. For instance, in our HER2 expression model, the AUC of 0.87 reflects high discriminative power, while DCA shows that applying the model at a 15–50% threshold range would prevent 35–50% of unnecessary biopsies without compromising sensitivity. While AUC-ROC quantifies diagnostic accuracy, DCA evaluates clinical net benefit. Both metrics were analyzed (**Figure 4D**), but AUC remains the gold standard for direct comparison with prior radiomics studies, such as those investigating RAD51, SCN3B, and CDK2 in cancer biomarker discovery (22–24).

Recent studies have focused on the relationship between breast cancer lymph node metastasis and primary lesion imaging features (25). While significant progress has been made, most radiomics research has focused on predicting non-sentinel lymph node (SLN) status (26) or axillary lymph node (ALN) (27, 28) metastasis to reduce postoperative complications. Research on SLN status, however, remains limited. This study found that ethnicity, lesion size, vascular tumor thrombus, clinical stage, mammography-based tumor margin, and HER2 expression were independent predictors of SLN metastasis, aligning with previous studies (4, 29). Previous research has highlighted a strong correlation between tumor margin characteristics and ALN metastasis (ALNM) risk (30), Spiculated margins, in particular, increase ALNM risk by approximately sixfold compared to clear margins, a finding supported by this study. This may be attributed to cancer cell infiltration inducing fibrosis, which accelerates the formation of blood and lymphatic vessels, thereby facilitating tumor spread. However, some studies suggest that fibroplasia might slightly delay tumor spread (31). HER2 is a transmembrane receptor protein with tyrosine kinase activity, typically in an inactive state, playing a role in cell growth and differentiation. HER2 positive is associated with tumor development and metastasis. This study confirmed the association between high HER2 expression and SLN metastasis, consistent with the findings of Ding J et al. (31). Other studies have also identified vascular invasion and tumor size as strongly correlated with SLN metastasis. In this study, vascular tumor thrombus was regarded as a key predictor; when present, the risk of SLN metastasis was 4.27 times higher than that of patients without vascular tumor thrombus, and the impact exceeded other indicators. This suggests that the tumor may have broken through the local limitations of the breast and has higher potential for spread and metastasis, further highlighting the value of clinical pathological factors in predicting SLN status and providing a strong basis for individualized treatment strategies. To explore a non-invasive and efficient method for identifying SLN status before surgery, the predictive model constructed based on mammography in this study achieved an area under the receiver operating characteristic (ROC) curve (AUC) of 0.84 in the training set. It is expected to serve as a digital biomarker that conveys information similar to SLN biopsy or lymph node dissection, providing an important reference for clinical treatment decisions. In comparison, Dong et al. (32) predicted lymph node status based on T2WI-FS and DWI sequence imagomics, with AUC =0.805; while Ding et al. (33) used DCE-MRI intratumoral and combined intratumoral and peritumoral radiomics models, with AUCs of 0.704 and 0.796. These results are broadly consistent with this study’s findings. This study also found that multiple mammography features and clinical pathology factors are independently related to SLN status, highlighting the potential value of mammography imaging as a non-invasive tool to identify SLN status in breast cancer patients. With the growing use of neoadjuvant systemic therapy (NAST), SLN biopsy is frequently performed after neoadjuvant therapy. In these cases, radiomics evaluation can assist in subsequent treatment decisions, particularly when therapy leads to downstaging.

The precise stratification of HER2 expression subtypes (HER2-zero, -low, -positive) is pivotal for tailoring ADC therapies. For example, HER2-low patients, once considered ineligible for HER2-targeted drugs, now represent a population with emerging therapeutic options. The DESTINY-Breast04 trial (10) demonstrated that DS-8201 significantly improves survival in HER2-low metastatic breast cancer. Our radiomics model, with an AUC of 0.84 for HER2-low identification, provides a non-invasive tool to preoperatively pinpoint these candidates, thereby avoiding undertreatment due to misclassification. Moreover, integrating radiomics with clinical factors supports can optimize treatment strategies and minimize surgical overtreatment. This study revealed that several clinicopathological factors were independently associated with changes in HER2 expression patterns. These factors, including lesion size, PR expression, menopausal status, axillary lymph node status, Ki67, CK5/6, and mammography calcification, predicted HER2 zero, low, and positive expression, in line with previous studies (20, 34–36). The observed association between calcification features and HER2 expression may reflect underlying biological processes. Calcification, caused by calcium deposition in breast tissue, often develops due to tissue ischemia and necrosis resulting from hypoxia and nutrient deficiency in rapidly growing tumors (37). HER2 positive is known to drive tumor proliferation and metabolic reprogramming, potentially leading to hypoxia-induced necrosis and subsequent calcium deposition in the tumor microenvironment (38). Additionally, HER2 signaling activates pathways such as PI3K/AKT and MAPK, which promote cellular stress and apoptosis, further contributing to dystrophic calcifications (39). Radiomic features capturing clustered or linear calcifications on mammography may thus serve as non-invasive indicators of HER2-driven tumor aggressiveness. This hypothesis aligns with prior studies demonstrating that the presence of microcalcifications strongly increased the likelihood of HER2 positive (36). Similarly, our study found that calcification is strongly associated with HER2 expression, with the risk of calcified lesions being 1.51 times higher than non-calcified lesions. Clustered or linearly distributed calcifications should raise particular concern among clinicians. Integrating these biological insights with radiomic models could enhance their utility in guiding targeted therapies. Additionally, CK5/6, a basal cell marker, serves as an indicator of tumor cell differentiation and plays a crucial role in classifying breast cancer subtypes and evaluating invasiveness. As a basal cell marker, CK5/6 reflects the differentiation status of tumor cells and plays an important role in breast cancer subtype classification and invasive assessment. This study found that CK5/6 positivity is more common in less differentiated and HER2-low expressing breast cancers, especially in basal-like subtypes, which show greater invasiveness and metastatic ability. These findings further emphasize the potential of combining mammography imaging with clinical pathology factors in improving HER2 expression models and provide a valuable basis for developing personalized treatment strategies. Studies have also shown that a predictive model based on mammography can effectively distinguish the three HER2 expression states in breast cancer. In the test set, the model’s AUCs for distinguishing between HER2 positive, HER2 low expression, and HER2 zero expression were 0.87, 0.82, and 0.85, respectively, which was superior to the previously reported single-parameter MRI radiomics method (40). For instance, Bian et al.’s (41) multi-parameter MRI-based imaging study had an AUC of 0.76 in distinguishing HER2 positive from HER2 negative, but when identifying tumors with low HER2 expression and zero HER2 expression, the AUC was only 0.71. In contrast, the mammography imaging model in this study can more accurately distinguish different expression states of HER2, showing greater diagnostic efficiency. Although IHC and FISH are standard methods for assessing HER2 expression, their limitations include lack of representativeness from a single sample and tumor heterogeneity. This study suggests that incorporating radiomics features into diagnostics can assist pathologists in achieving more comprehensive HER2 identification and enhancing the precision of biopsy target selection (42, 43). Additionally, during neoadjuvant chemotherapy, radiomics can dynamically track HER2 expression changes, enabling timely adjustments to treatment strategies. For patients with drug-resistant or triple-negative breast cancer, imaging-guided re-detection of low HER2 expression in clinical trials may become a critical strategy for optimizing treatment. In addition, Future research may incorporate advanced nanomaterials to enhance imaging resolution and therapeutic monitoring, thereby refining radiomic feature extraction and clinical applicability (44).

Although our model demonstrated high diagnostic accuracy and potential clinical application value, its clinical translation requires validation in multicenter cohorts. We recognize that variations in mammography equipment and regional differences in HER2 testing protocols (e.g., IHC/FISH criteria) may impact model generalizability. To address this, we are attempting to initiate partnerships with institutions in geographically diverse regions of China, aiming to collect heterogeneous data for external validation.

## 5 Conclusion

In conclusion, breast cancer mammography radiomics demonstrated high accuracy in identifying HER2 subtypes and predicting sentinel lymph node (SLN) metastasis. This has significant implications for developing personalized treatment plans, assessing prognosis, and guiding clinical decision-making. However, the use of radiomics is still in its early stages. As data sharing expands and machine learning technology advances, its potential value in the medical field requires further exploration.

## 6 Limitation

This study has several limitations: (1) This study is limited by its single-center retrospective design, which may restrict the generalizability of the model to other populations. While we employed rigorous internal validation, future multicenter studies are imperative to assess performance across diverse ethnic groups, imaging devices, and clinical protocols. Challenges such as inter-institutional data harmonization and ethical approvals currently hinder immediate expansion, but collaborative efforts are underway; (2) ROI delineation was performed using a two-dimensional approach, which may be influenced by the volume effect. Future studies could consider using three-dimensional imaging to enhance accuracy; (3) Some clinical characteristics were assessed semi-qualitatively, and the results could be influenced by evaluator subjectivity.

## Data Availability

The raw data supporting the conclusions of this article will be made available by the authors, without undue reservation.

## References

[1] Siegel RL Giaquinto AN Jemal A. Cancer statistics, 2024. CA Cancer J Clin. (2024) 74:12–49. doi: 10.3322/caac.21820 38230766

[2] Sonkin D Thomas A Teicher Cancer treatments BA. Past, present, and future. Cancer Genet. (2024) 286-287:18–24. doi: 10.1016/j.cancergen.2024.06.002 38909530 PMC11338712

[3] Joshi RM Telang B Soni G Khalife A. Overview of perspectives on cancer, newer therapies, and future directions. Endoscopic Ultrasound. (2024) 10:105–9. doi: 10.1097/ot9.0000000000000039

[4] Marino MA Avendano D Zapata P Riedl CC Pinker K. Lymph node imaging in patients with primary breast cancer: concurrent diagnostic tools. Oncologist. (2020) 25:e231–42. doi: 10.1634/theoncologist.2019-0427 PMC7011661 32043792

[5] Zha HL Zong M Liu XP Pan JZ Wang H Gong HY. Preoperative ultrasound-based radiomics score can improve the accuracy of the Memorial Sloan Kettering Cancer Center nomogram for predicting sentinel lymph node metastasis in breast cancer. Eur J Radiol. (2021) 135:109512. doi: 10.1016/j.ejrad.2020.109512 33429302

[6] Harbeck N Penault-Llorca F Cortes J Gnant M Houssami N Poortmans P. Breast cancer. Nat Rev Dis Primers. (2019) 5:66. doi: 10.1038/s41572-019-0111-2 31548545

[7] Zou Y Zheng S Xie X Ye F Hu X Tian Z. N6-methyladenosine regulated FGFR4 attenuates ferroptotic cell death in recalcitrant HER2-positive breast cancer. Nat Commun. (2022) 13:2672. doi: 10.1038/s41467-022-30217-7 35562334 PMC9106694

[8] Agostinetto E Rediti M Fimereli D Debien V Piccart M Aftimos P. HER2-low breast cancer: molecular characteristics and prognosis. Cancers (Basel). (2021) 13:11–2. doi: 10.3390/cancers13112824 PMC8201345 34198891

[9] Shao X Xie N Chen Z Wang X Cao W Zheng Y. Inetetamab for injection in combination with vinorelbine weekly or every three weeks in HER2-positive metastatic breast cancer: A multicenter, randomized, phase II clinical trial. J Transl Int Med. (2024) 12:466–77. doi: 10.1515/jtim-2024-0022 PMC11538898 39513033

[10] Modi S Jacot W Yamashita T Sohn J Vidal M Tokunaga E. Trastuzumab deruxtecan in previously treated HER2-low advanced breast cancer. N Engl J Med. (2022) 387:9–20. doi: 10.1056/NEJMoa2203690 35665782 PMC10561652

[11] Eiger D Agostinetto E Saúde-Conde R de Azambuja E. The exciting new field of HER2-Low breast cancer treatment. Cancers (Basel). (2021) 13:1–3. doi: 10.3390/cancers13051015 PMC7957750 33804398

[12] Eun NL Kang D Son EJ Park JS Youk JH Kim JA. Texture analysis with 3.0-T MRI for association of response to neoadjuvant chemotherapy in breast cancer. Radiology. (2020) 294:31–41. doi: 10.1148/radiol.2019182718 31769740

[13] Chang JM Leung JWT Moy L Ha SM Moon WK. Axillary nodal evaluation in breast cancer: state of the art. Radiology. (2020) 295:500–15. doi: 10.1148/radiol.2020192534 32315268

[14] Afrin H Larson NB Fatemi M Alizad A. Deep learning in different ultrasound methods for breast cancer, from diagnosis to prognosis: current trends, challenges, and an analysis. Cancers (Basel). (2023) 15:5–6. doi: 10.3390/cancers15123139 PMC10296633 37370748

[15] Song X Xu H Wang X Liu W Leng X Hu Y. Use of ultrasound imaging Omics in predicting molecular typing and assessing the risk of postoperative recurrence in breast cancer. BMC Womens Health. (2024) 24:380. doi: 10.1186/s12905-024-03231-8 38956552 PMC11218367

[16] Xu Z Yang Q Li M Gu J Du C Chen Y. Predicting HER2 status in breast cancer on ultrasound images using deep learning method. Front Oncol. (2022) 12:829041. doi: 10.3389/fonc.2022.829041 35251999 PMC8889619

[17] Quan MY Huang YX Wang CY Zhang Q Chang C. and S C Zhou Deep learning radiomics model based on breast ultrasound video to predict HER2 expression status. Front Endocrinol (Lausanne). (2023) 14:1144812. doi: 10.3389/fendo.2023.1144812 37143737 PMC10153672

[18] Huang Y Wei L Hu Y Shao N Lin Y He S. Multi-parametric MRI-based radiomics models for predicting molecular subtype and androgen receptor expression in breast cancer. Front Oncol. (2021) 11:706733. doi: 10.3389/fonc.2021.706733 34490107 PMC8416497

[19] Wolff AC Hammond MEH Allison KH Harvey BE Mangu PB Bartlett JMS. Human epidermal growth factor receptor 2 testing in breast cancer: American society of clinical oncology/college of American pathologists clinical practice guideline focused update. J Clin Oncol. (2018) 36:2105–22. doi: 10.1200/jco.2018.77.8738 29846122

[20] Ramtohul T Djerroudi L Lissavalid E Nhy C Redon L Ikni L. Multiparametric MRI and radiomics for the prediction of HER2-zero, -low, and -positive breast cancers. Radiology. (2023) 308:e222646. doi: 10.1148/radiol.222646 37526540

[21] Chen M Kong C Lin G Chen W Guo X Chen Y. Development and validation of convolutional neural network-based model to predict the risk of sentinel or non-sentinel lymph node metastasis in patients with breast cancer: a machine learning study. EClinicalMedicine. (2023) 63:102176. doi: 10.1016/j.eclinm.2023.102176 37662514 PMC10474371

[22] Liu H Weng J Huang CL Jackson AP. Is the voltage-gated sodium channel β3 subunit (SCN3B) a biomarker for glioma? Funct Integr Genomics. (2024) 24:162. doi: 10.1007/s10142-024-01443-7 39289188

[23] Liu H Weng J. A pan-cancer bioinformatic analysis of RAD51 regarding the values for diagnosis, prognosis, and therapeutic prediction. Front Oncol. (2022) 12:858756. doi: 10.3389/fonc.2022.858756 35359409 PMC8960930

[24] Liu H Weng J. A comprehensive bioinformatic analysis of cyclin-dependent kinase 2 (CDK2) in glioma. Gene. (2022) 822:146325. doi: 10.1016/j.gene.2022.146325 35183683

[25] Wang C Chen X Luo H Liu Y Meng R Wang M. Development and internal validation of a preoperative prediction model for sentinel lymph node status in breast cancer: combining radiomics signature and clinical factors. Front Oncol. (2021) 11:754843. doi: 10.3389/fonc.2021.754843 34820327 PMC8606782

[26] Qiu Y Zhang X Wu Z Wu S Yang Z Wang D. MRI-based radiomics nomogram: prediction of axillary non-sentinel lymph node metastasis in patients with sentinel lymph node-positive breast cancer. Front Oncol. (2022) 12:811347. doi: 10.3389/fonc.2022.811347 35296027 PMC8920306

[27] Fong W Tan L Tan C Wang H Liu F Tian H. Predicting the risk of axillary lymph node metastasis in early breast cancer patients based on ultrasonographic-clinicopathologic features and the use of nomograms: a prospective single-center observational study. Eur Radiol. (2022) 32:8200–12. doi: 10.1007/s00330-022-08855-8 36169686

[28] Yu Y Tan Y Xie C Hu Q Ouyang J Chen Y. Development and validation of a preoperative magnetic resonance imaging radiomics-based signature to predict axillary lymph node metastasis and disease-free survival in patients with early-stage breast cancer. JAMA Netw Open. (2020) 3:e2028086. doi: 10.1001/jamanetworkopen.2020.28086 33289845 PMC7724560

[29] Liu L Lin Y Li G Zhang L Zhang X Wu J. A novel nomogram for decision-making assistance on exemption of axillary lymph node dissection in T1–2 breast cancer with only one sentinel lymph node metastasis. Front Oncol. (2022) 12:924298. doi: 10.3389/fonc.2022.924298 36172144 PMC9511144

[30] Zong Q Deng J Ge W Chen J Xu D. Establishment of simple nomograms for predicting axillary lymph node involvement in early breast cancer. Cancer Manag Res. (2020) 12:2025–35. doi: 10.2147/cmar.S241641 PMC7090154 32256110

[31] Eun NL Bae SJ Youk JH Son EJ Ahn SG Jeong J. Tumor-infiltrating lymphocyte level consistently correlates with lower stiffness measured by shear-wave elastography: subtype-specific analysis of its implication in breast cancer. Cancers (Basel). (2024) 16:9–10. doi: 10.3390/cancers16071254 PMC11011118 38610934

[32] Liu Y Li X Zhu L Zhao Z Wang T Zhang X. Preoperative prediction of axillary lymph node metastasis in breast cancer based on intratumoral and peritumoral DCE-MRI radiomics nomogram. Contrast Media Mol Imaging. (2022) 2022:6729473. doi: 10.1155/2022/6729473 36051932 PMC9410821

[33] Ding J Chen S Serrano Sosa M Cattell R Lei L Sun J. Optimizing the peritumoral region size in radiomics analysis for sentinel lymph node status prediction in breast cancer. Acad Radiol. (2022) 29 Suppl 1:S223–s228. doi: 10.1016/j.acra.2020.10.015 33160860 PMC9583077

[34] Yin Y Mo S Li G Wu H Hu J Zheng J. Ultrasound radiomics for the prediction of breast cancers with HER2-zero, -low, and -positive status: A dual-center study. Technol Cancer Res Treat. (2024) 23:15330338241292668. doi: 10.1177/15330338241292668 39470030 PMC11526407

[35] Xu A Chu X Zhang S Zheng J Shi D Lv S. Prediction breast molecular typing of invasive ductal carcinoma based on dynamic contrast enhancement magnetic resonance imaging radiomics characteristics: A feasibility study. Front Oncol. (2022) 12:799232. doi: 10.3389/fonc.2022.799232 35664741 PMC9160981

[36] Deng Y Lu Y Li X Zhu Y Zhao Y Ruan Z. Prediction of human epidermal growth factor receptor 2 (HER2) status in breast cancer by mammographic radiomics features and clinical characteristics: a multicenter study. Eur Radiol. (2024) 34:5464–76. doi: 10.1007/s00330-024-10607-9 38276982

[37] Azam S Eriksson M Sjölander A Gabrielson M Hellgren R Czene K. Mammographic microcalcifications and risk of breast cancer. Br J Cancer. (2021) 125:759–65. doi: 10.1038/s41416-021-01459-x PMC8405644 34127810

[38] O’Grady S. and M P Morgan Microcalcifications in breast cancer: From pathophysiology to diagnosis and prognosis. Biochim Biophys Acta Rev Cancer. (2018) 1869:310–20. doi: 10.1016/j.bbcan.2018.04.006 29684522

[39] Miricescu D Totan A Stanescu-Spinu II Badoiu SC Stefani C Greabu M. PI3K/AKT/mTOR signaling pathway in breast cancer: from molecular landscape to clinical aspects. Int J Mol Sci. (2020) 22:4–10. doi: 10.3390/ijms22010173 PMC7796017 33375317

[40] Xu A Chu X Zhang S Zheng J Shi D Lv S. Development and validation of a clinicoradiomic nomogram to assess the HER2 status of patients with invasive ductal carcinoma. BMC Cancer. (2022) 22:872. doi: 10.1186/s12885-022-09967-6 35945526 PMC9364617

[41] Bian X Du S Yue Z Gao S Zhao R Huang G. Potential antihuman epidermal growth factor receptor 2 target therapy beneficiaries: the role of MRI-based radiomics in distinguishing human epidermal growth factor receptor 2-low status of breast cancer. J Magn Reson Imaging. (2023) 58:1603–14. doi: 10.1002/jmri.28628 36763035

[42] Zheng S Yang Z Du G Zhang Y Jiang C Xu T. Discrimination between HER2-overexpressing, -low-expressing, and -zero-expressing statuses in breast cancer using multiparametric MRI-based radiomics. Eur Radiol. (2024) 34:6132–44. doi: 10.1007/s00330-024-10641-7 38363315

[43] Peng Y Zhang X Qiu Y Li B Yang Z Huang J. Development and validation of MRI radiomics models to differentiate HER2-zero, -low, and -positive breast cancer. AJR Am J Roentgenol. (2024) 222:e2330603. doi: 10.2214/ajr.23.30603 38265001

[44] Zhou M Tian M Li C. Copper-based nanomaterials for cancer imaging and therapy. Bioconjug Chem. (2016) 27:1188–99. doi: 10.1021/acs.bioconjchem.6b00156 27094828

